# PacBio full-length sequencing integrated with RNA-seq reveals the molecular mechanism of waterlogging and its recovery in *Paeonia ostii*

**DOI:** 10.3389/fpls.2022.1030584

**Published:** 2022-11-03

**Authors:** Xiaoxiao Zhang, Xiang Liu, Minghui Zhou, Yonghong Hu, Junhui Yuan

**Affiliations:** ^1^ College of Landscape Architecture and Arts, Northwest A&F University, Yangling, Shaanxi, China; ^2^ Shanghai Key Laboratory of Plant Functional Genomics and Resources, Shanghai Chenshan Botanical Garden, Shanghai, China; ^3^ School of Ecological Technology and Engineering, Shanghai Institute of Technology, Shanghai, China

**Keywords:** tree peony, transcriptome, waterlogging, waterlogging recovery, hypoxia

## Abstract

*Paeonia ostii*, a widely cultivated tree peony species in China, is a resourceful plant with medicinal, ornamental and oil value. However, fleshy roots lead to a low tolerance to waterlogging in *P. ostii*. In this study, *P. ostii* roots were sequenced using a hybrid approach combining single-molecule real-time and next-generation sequencing platforms to understand the molecular mechanism underlying the response to this sequentially waterlogging stress, the normal growth, waterlogging treatment (WT), and waterlogging recovery treatment (WRT). Our results indicated that the strategy of *P. ostii*, in response to WT, was a hypoxic resting syndrome, wherein the glycolysis and fermentation processes were accelerated to maintain energy levels and the tricarboxylic acid cycle was inhibited. *P. ostii* enhanced waterlogging tolerance by reducing the uptake of nitrate and water from the soil. Moreover, transcription factors, such as AP2/EREBP, WRKY, MYB, and NAC, played essential roles in response to WT and WRT. They were all induced in response to the WT condition, while the decreasing expression levels were observed under the WRT condition. Our results contribute to understanding the defense mechanisms against waterlogging stress in *P. ostii*.

## Introduction

*Paeonia ostii*, belonging to section *Moutan* DC. of the genus *Paeonia* L. (Paeoniaceae), is a perennial woody shrub native to China (Zhang et al., 2017a). Paeonia *ostii* has been cultivated as ornamental and medicinal in China for more than 1500 years (Li et al., 2011; Hu and Han, 2018). In recent years, *P. ostii* was planted extensively to harvest the roots for the traditional Chinese medicine “Mudanpi” with functions of clearing heat and cooling blood, while recently, farmers have also started collecting the seeds for oil production (Hu and Han, 2018). It has been found that the seeds of *P. ostii* are rich in unsaturated fatty acids, especially *α*-linolenic acid (Li et al., 2015; Yu et al., 2016), which are beneficial for human health (Lefevre et al., 2004; Shahidi and Miraliakbari, 2005). Seed oil has been authenticated as a new resource of functional food since 2011 (Zhang et al., 2017b). Moreover, the area under cultivation of *P. ostii* is the largest among all the ten tree peony species and nearly 1500 cultivars in China (Zhang et al., 2018a), with the flower being white and monopetalous. In the middle and lower reaches of the Yangtze River, an irregular spatial and temporal distribution of precipitation frequently occurs, which results in water levels that exceed the requirement for *P. ostii*, causing waterlogging in the field (Ding et al., 2020). Therefore, *P. ostii* often fails to grow well in this region, and the main reasons for this phenomenon are waterlogging and high temperature (Wu et al., 2009; Hu et al., 2017).

Waterlogging can be defined as the saturation of soils with water, and it often occurs during the rainy season. Waterlogging is an adverse abiotic stress that limits the oxygen diffusion into the soil and creates a low oxygen (hypoxia) environment around the plant roots (Juntawong et al., 2014). Hypoxia can accelerate the anaerobic respiration, reduce the root activity, and result in energy shortage. Plants can temporarily maintain energy production to some extent during hypoxia caused by waterlogging, *via* glycolysis and ethanol fermentation (Pan et al., 2021). The genes encoding enzymes involved in these pathways such as pyruvate decarboxylase (PDC), alcohol dehydrogenase (ADH), and lactate dehydrogenase (LDH) were consistently upregulated under waterlogging stress (Yin et al., 2019). Besides, a key feature for the acclimation to hypoxia is to activate genes encoding enzymes involved in transcription regulation and signaling pathways in order to allow biological and physiological adjustments to the hypoxia conditions (Bailey-Serres et al., 2012). The ethylene response factor represented the highest number of significantly expressed TFs under hypoxic conditions followed by bHLH, MYB, NAC, and WRKY in soybean (Chen et al., 2016). As waterlogging recedes, these plant roots, which had adjusted to the reduced light and oxygen in murky water, are suddenly re-exposed to aerial conditions. The shift to an intensely reoxygenated environment poses an additional stress for the plant, namely oxidative stress. Therefore, waterlogging acts as sequential stress manipulating plant growth (Yeung et al., 2018). Our previous study showed that the physiological response to waterlogging of *P. ostii* was rapid and sensitive. Despite its failure to tolerate long-term waterlogging, *P. ostii* possesses a strong recovery ability in the critical frame (within 72 h after waterlogging) (Hu et al., 2017). However, the molecular mechanisms underlying the responses to waterlogging and waterlogging recovery in *P. ostii* remain largely unexplored.

As a next-generation sequencing (NGS) technique, RNA-sequencing has become an indispensable tool for transcriptome-wide analysis of differential gene expression and gene regulatory networks (Hu et al., 2020). RNA-seq provides a precise and comprehensive analysis of RNA transcripts that affect gene expression (Chang et al., 2019). It has recently been widely used for researching *P. ostii* gene expression in response to various stresses, including chill-induced stress (Gai et al., 2013), copper stress (Wang et al., 2016), and drought-induced stress (Zhao et al., 2019). However, for the species without reference genomes, NGS was insufficient to analyze their gene expression because of inaccurate reconstruction and lack of reliable expression estimation of transcript variants due to the short, sequenced reads (Steijger et al., 2013). Single-molecule real-time (SMRT) sequencing, a third-generation technique developed by Pacific Biosciences (PacBio) to sequence cDNA, can produce longer reads than NGS and avoids further assembly (Sharon et al., 2013). The combination of SMRT and NGS provided researchers with an efficient and effective way to determine gene expression patterns, thereby exploring unanswered biological questions, particularly for species lacking genome-related information (Minoche et al., 2015; Deng et al., 2018; Jia et al., 2018).

Plant roots are the tissues in direct contact with water under waterlogging stress. Thus, we hypothesized that identifying the genes upregulated and downregulated under hypoxia in *P. ostii* roots can deepen our understanding of the molecular regulatory pathways in response to waterlogging and waterlogging recovery. In the present study, the seedings with 3 d of waterlogging treatment and followed by a 7-d recovery treatment, were selected as subjects, according to our previous results (Hu et al., 2017). We constructed a *de novo* full-length transcriptomic database for seedlings roots under all conditions using the SMRT sequencing. Furthermore, RNA-seq was adopted to identify the differentially expressed genes (DEGs) and analyze the specific pathways involved in waterlogging and waterlogging recovery treatment. Thus, the results of this study will deepen our understanding of the defense mechanisms against waterlogging stress in *P. ostii* and provide theoretical support for the molecular breeding of *P. ostii* to withstand waterlogging stress.

## Materials and methods

### Plant materials

In the present study, 3-year-old potted *P. ostii* were used as study materials. On March 1, the well-grown plants cultivated in the field were transplanted into the pots, and then the plants were transported to the phytotron. The day and night temperatures were set to (22 ± 1)°C and (16 ± 1)°C, respectively, with a photoperiod of 16 h light/8 h dark, 60% relative humidity, 40% soil water content, and light intensity of 600 μmol m^-2^ s^-1^. On May 1, uniform plants were selected and randomly divided into three groups. In the control (CK) group for waterlogging treatment (WT), the sampling time was 0 h post-waterlogging. The WT water level was set at 2 cm above the soil, and the sampling time was 3 d after waterlogging. For the waterlogging recovery treatment (WRT), the soil surface water was removed after 3 d of waterlogging, and the sampling time was 7 d after recovery. Then, the same plants from the WT group were also used as the control for the WRT group. For RNA-seq, two biological replicates were performed, and each biological sample consisted of the roots of three uniform plants. The mixed samples from these three sampling points were collected for full-length sequencing. All these samples were immediately frozen in liquid nitrogen and stored at -80°C until RNA extraction. Total RNA was extracted from the roots using TIANGEN RNA Prep Pure Plant Plus kit (Tiangen Biotech Co. Ltd., Beijing, China), and the quality and quantity of RNA were assessed using an Agilent Bioanalyzer 2100 (Agilent Technologies, Santa Clara, CA, USA).

### Measurement of morphological and physiological indices

The roots of plants from three treatments were washed with deionized water, and root morphology was photographed by Epson Perfection V700 Photo (Epson (China) Co., Ltd., China). Root tips were cut by a knife blade, fixed in FAA, and then conducted by using a Saffron-O and Fast Green Stain Kit (Solarbio, Beijing, China) based on the manufacturers’ instructions. The cell morphology of root tip was viewed with an optical microscope (BX43, Olympus, Tokyo, Japan). Root activity was detected by triphenyl tetrazolium chloride (TTC) reduction method (Hu et al., 2017). Relative electrical conductivity (REC) of the root was measured as described previously (Zhao et al., 2019). All tests were performed with three biological replicates.

### Illumina transcriptome library preparation and sequencing

A total of 5 μg RNA per sample was used as input material to generate sequencing libraries using the NEBNext^®^ Ultra™ RNA Library Prep Kit for Illumina^®^ (NEB, USA) following the manufacturer’s recommendations. Briefly, mRNA was purified from total RNA using poly-T oligo-attached magnetic beads. Fragmentation was carried out in the NEBNext First Strand Synthesis Reaction Buffer (5X). First strand cDNA was synthesized using random hexamer primer and M-MuLV Reverse Transcriptase (RNase H-). Second strand cDNA synthesis was subsequently performed using DNA polymerase I and RNase H. Remaining overhangs were converted into blunt ends *via* exonuclease/polymerase activities. After adenylation of 3’ ends of DNA fragments, NEBNext Adaptors with hairpin loop structures were ligated to prepare for hybridization. The library fragments were purified with the AMPure XP system (Beckman Coulter, Beverly, USA) to identify cDNA fragments of about 300 bp length. Then, 3 μl USER Enzyme (NEB, USA) was incubated with size-selected, adaptor-ligated cDNA at 37°C for 15 min followed by 5 min at 95°C. Then PCR was performed with Phusion^®^ High-Fidelity DNA polymerase, Universal PCR primers and Index (X) Primer. Finally, PCR products were purified with AMPure XP system and library quality was assessed on the Agilent Bioanalyzer 2100 system. The clustering of index-coded samples was performed on a cBot Cluster Generation System using the TruSeq PE Cluster Kit v3-cBot-HS (Illumina) according to the manufacturer’s instructions. After cluster generation, the libraries were sequenced on an Illumina Hiseq 2500 platform, and paired-end reads were generated.

### PacBio SMRT bell library preparation and sequencing

The Iso-seq template was prepared based on the protocol of Iso-Seq Template Preparation for Sequel Systems. First strand cDNA was synthesized from 800–1000 ng total RNA using Clontech SMARTer™ PCR cDNA Synthesis Kit (Clontech Laboratories, Inc., USA). The CDS Primer IIA was first annealed to the polyA+ tail of transcripts, followed by first-strand synthesis with SMARTScribe™ Reverse Transcriptase. Then, a sufficient amount of double-stranded cDNA was produced using large-scale PCR with Clontech PrimeSTAR GXL DNA Polymerase and 5’PCR Primer IIA (5’- AAGCAGTGGTATCAACGCAGAGTAC-3’). After PCR amplification, the products were selected using the BluePippin Size Selection System with the following bins for each sample: 1–2, 2–3, and > 3 kb. The DNA was repaired by DNA Damage Repair Mix (PacBio) and End Repair Mix (PacBio). Blunt Adapter was ligated to the cDNA using the ligase from PacBio. Exonucleases Exo III and EXO VII were added to the ligated cDNA, to reduce the amount of unrepaired DNA or linear DNA without a blunt adapter. Finally, the obtained cDNAs were measured using Qubit HS (Life Technologies, USA) and Agilent Bioanalyzer 2100. Sequencing reactions were performed using the PacBio Sequel sequencer (BGI-Shenzhen, China) with Sequel Sequencing Kit 2.1 and Sequel SMRT Cell 1M v2 Tray.

### Data processing of PacBio sequencing reads

Raw sequencing data (also called raw polymerase reads) produced by the Pacific Biosciences Sequel system were processed following the IsoSeq protocol through the SMRT analysis package version 2.3.0 (Pacific Biosciences, https://www.pacb.com/products-and-services/analytical-software/smrt-analysis). These data have been deposited in the Genome Sequence Archive (https://bigd.big.ac.cn/gsa) in the National Genomics Data Center, under the accession number: CRA004521. The raw polymerase reads that had full passes >0 and the predicted consensus accuracy >0.75 were selected for producing ROIs (Reads of Insert). ROIs with a minimum length of 300 bp were classified into full-length and non-full-length transcript sequences, based on whether the 5’ primer, 3’ primer, and poly-A tail were all observed. The full-length sequences were processed to *de novo* consensus isoforms using the Iterative Clustering for Error Correction algorithm and then polished *via* the Quvier quality-aware algorithm. The *de novo* consensus isoforms with high quality (the expected Quiver accuracy ≥ 0.95) from each library were merged, and redundancy was removed using Cluster Database at High Identity with Tolerance (CD-HIT) (Fu et al., 2012) based on the sequence similarity, to obtain final unique full-length isoforms. The coding sequences of the isoforms were identified by Transdecoder (v3.0.1), and then the longest one was selected perform BLAST (Basic Local Alignment Search Tool).

### Functional annotation

Final full-length isoforms were mapped to the NCBI non-redundant protein sequences (NR), NCBI non-redundant nucleotide sequence (NT), SwissProt (a manually annotated and reviewed protein sequence database), Kyoto Encyclopedia of Genes and Genomes (version 59) (KEGG), and Clusters of Eukaryotic Orthologous Groups (KOG) database by Blast software (version 2.2.23, https://blast.ncbi.nlm.nih.gov/Blast.cgi) (Altschul et al., 1990) with default parameters (under a threshold E-value ≤10^-5^) to get the isoform annotations. Gene Ontology (GO) annotations and functional classifications were obtained using the Blast2GO program (version 2.5.0, E-value ≤10^-5^, https://www.blast2go.com) (Conesa et al., 2005) based on NR annotations. Hmmscan software (version 5.11- 51.0) (Jones et al., 2014) as used to obtain the annotations from the Pfam database.

### Illumina data analysis

The sequencing data was filtered with SOAPnuke (v1.5.2, https://github.com/BGI-flexlab/SOAPnuke) (Li et al., 2008), and clean reads were obtained and stored in FASTQ format. The clean reads were mapped to reference full-length transcriptome using HISAT2 (v2.0.4, http://www.ccb.jhu.edu/software/hisat/index.shtml) (Kim et al., 2015). Bowtie2 (v2.2.5, http://bowtiebio.sourceforge.net/%20Bowtie2%20/index.shtml) (Langmead and Salzberg, 2012) was applied to align the clean reads to the reference coding gene set and then the expression levels of genes were calculated using RSEM (v1.2.12, https://github.com/deweylab/RSEM) (Li and Dewey, 2011). Essentially, differential expression analysis was performed using the DESeq2 (v1.4.5, http://www.bioconductor.org/packages/release/bioc/html/DESeq2.html) with Q value ≤ 0.05. DEGs were identified using DESeq2 (Love et al., 2014) with Q value (adjust P value) < 0.001 and fold change ≥ 2 or ≤ -2. The identified DEGs were subsequently subjected to GO and KEGG enrichment using Phyper in the R package, with default Q value <= 0.05.

### Quantitative real-time PCR (qRT-PCR)

First-strand cDNA was prepared from 1 μg of total RNA per sample, using a FastKing RT Kit with gDNase (Tiangen Biotech Co. Ltd., Beijing, China). Specific primers were designed for each of the 14 selected isoforms (**Table S1**). PCRs were performed on an ABI StepOnePlus^®^ Real-Time PCR System (Applied Biosystems, California, USA), following the manufacturer’s instructions. Each reaction mixture (20 μl) contained 10 μl of TB Green Premix Ex Taq II (Tli RNaseH Plus) (Takara), 0.8 μl of each primer (10 μM), 0.3 μl of cDNA template (1 μg), and 8.1 μl of RNase-free water. PCR for each gene was performed in triplicate, with the following thermal cycling conditions: 95°C for 30 s; 40 cycles of 95°C for 5 s and 64°C for 30s; and 95°C for 15 s. Primer specificity was confirmed *via* melt curve analysis. The relative expression levels of the tested genes were calculated *via* the 2^-ΔΔCt^ method, using the *β*-*actin* gene as internal controls.

### Statistical analysis

The results were expressed as the mean ± standard deviation (SD). One-way analysis of variance (ANOVA) and Duncan’s multiple range tests were used to analyze the significance at a *p* level of 0.05 by SPSS software (version 19.0 for Windows; SPSS Inc., 2010). The gene expression heatmap was performed using the OmicShare tools (https://www.omicshare.com/tools).

## Results

### Morphological and physiological indices

**Figure 1:**
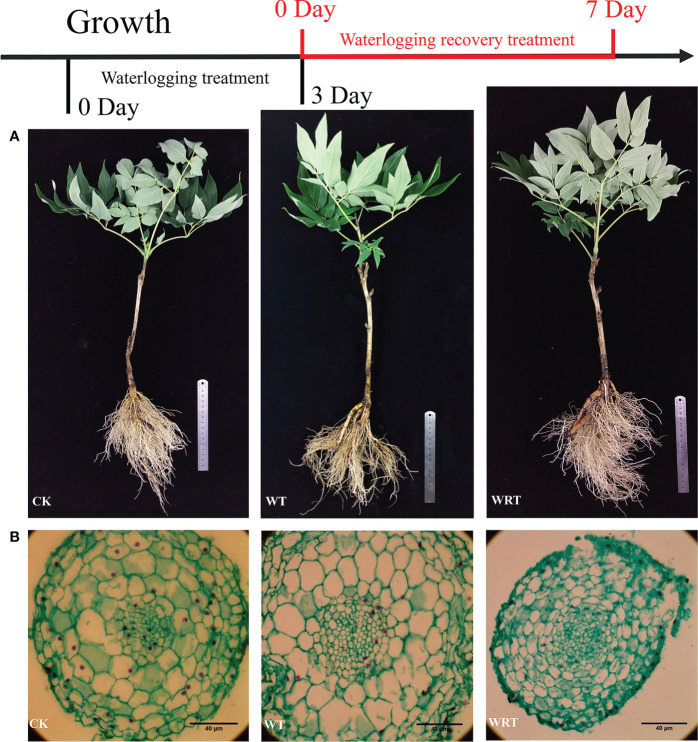
Morphological changes of the control (CK), waterlogging treatment (WT), and waterlogging recovery treatment (WRT) of *P. ostii.* and anatomic **(A)**, Root morphology; **(B)**, Cell morphology of root tip.

As shown in **Figure 1A**, the fibrous roots of plants in CK group were numerous and white. After waterlogging, almost half of the fibrous roots fell off, and the remaining fibrous roots became brown. The tips of some main roots were rotten. For the WRT group, although the rot of main root was serious, more fibrous roots had developed. These new fibrous roots were white, and the root system was basically the same as that of CK group. In terms of the anatomy of root tip cell (**Figure 1B**), the size became large after waterlogging, but the cytoplasm in most cells decreased. For the WRT group, the cell size of root tip decreased, and the cytoplasm in most cells increased.

**Figure 2:**
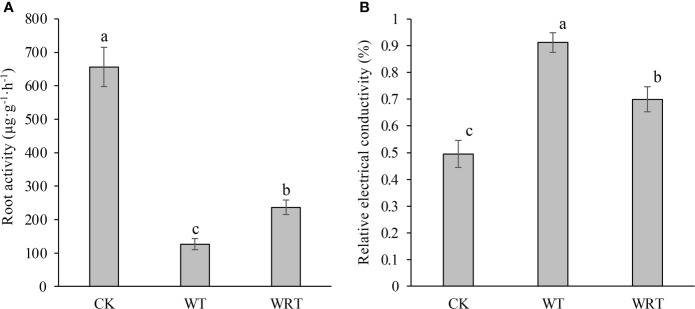
Physiological changes of the control (CK), waterlogging treatment (WT), and waterlogging recovery treatment (WRT) of *P. ostii.* **(A)**, Root activity; **(B)**, Relative electrical conductivity of root. Values represent mean ± standard deviation (SD), and letters indicate significant differences according to Duncan’s multiple range test (*p* < 0.05).

The plant in the CK group had high root activity (**Figure 2A**). After waterlogging, the root activity decreased significantly, and the activity level was about one sixth of that of CK group. When the soil surface water was removed, the root activity slightly recovered. Although the root activity of plants in WRT group had reached about twice that of WT group, it was still significantly lower than that of CK group. An opposite trend was observed in the REC of plant root under different treatments (**Figure 2B**). There was significant variation in the REC of plant root among three treatment groups, and the highest value was found in the root of WT group.

### PacBio ISO-seq analysis

PacBio ISO-seq was used in this study to sequence the mixed root samples and to construct a *de novo* full-length transcriptomic database due to the lack of a *P. ostii* reference genome. Moreover, 11 cDNA libraries, including insert fragments of 1–2 kb, 2–3 kb, and >3 kb, were prepared. In summary, 1,272,956 polymerase reads were generated by the PacBio Sequel platform (**Table S2**). Polymerase reads with full passes > 0 and the predicted consensus accuracy > 0.75 were selected for producing ROIs, and 1,055,458 ROIs were obtained in all libraries (**Table S3**). Furthermore, 546,928 full-length non-chimeric (FLNC) reads with poly (A) tail signals, 5’ adaptor sequences, and 3’ adapter sequences were obtained (**Table S4**). According to the isoform-level clustering algorithm, the full-length sequences were processed to *de novo* consensus isoforms. The isoforms were polished using Quvier quality-aware algorithm. The results from each library were merged, and redundancy was removed using CD-HIT. Finally, the transcripts contained 187,564 unique full-length isoforms, and those with an average length of 2323 bp were considered in the reference transcriptome and used for further analysis (**Table S5**). We further evaluated the completeness of the assemblies by comparing them against a set of conserved plant genes in the Benchmarking Universal Single-Copy Orthologs (BUSCO, embryophyta_odb9 dataset) using BUSCO v2.0 pipeline. The complete sequence accounted for 89.77% of all sequences (**Figure S1A**).

### Annotation of the full-length reference transcriptome

All the isoforms of the reference transcriptome were aligned to the public databases including NCBI non-redundant proteins (NR), NCBI non-redundant nucleotides (NT), SwissProt, Kyoto Encyclopedia of Genes and Genomes (KEGG), KOG (Clusters of Eukaryotic Orthologous Groups), Pfam, and Gene Ontology (GO) (**Table 1**). Our results indicated that a total of 156,402 isoforms (83.39%) were annotated in at least one database, and 48,392 isoforms (25.80%) had significant matches in all seven databases. The best-harbored isoforms in the reference transcriptome were in the NR database (147,828, 78.81%), while only 85,754 isoforms (45.72%) got hits in the Pfam database. Overall, 59,824 isoforms got hits in five databases (NR, KEGG, KOG, SwissProt, and Pfam) (**Figure S1B**).

**Table 1:** Information of function annotation.

Database	Annotated Number	Percentage
Nr	147,828	78.81%
Nt	117,845	62.83%
SwissProt	108,136	57.65%
KEGG	118,052	62.94%
KOG	119,842	63.89%
Pfam	85,754	45.72%
GO	115,289	61.47%
Intersection	48,392	25.80%

For GO analysis, the isoforms were assigned to 54 functional groups, which were allocated into three ontologies: biological process (167,903 isoforms), cellular component (230,396 isoforms), and molecular function (146,236 isoforms) (**Figure S1C**). For the biological process category, isoforms involved in cellular processes (GO:0009987, 50,400 isoforms) and metabolic processes (GO:0008152, 45,217 isoforms) were highly represented. Cell (GO:0005623, 44,564 isoforms), cell part (GO:0044464, 43,542 isoforms), membrane (GO:0016020, 36,727 isoforms), organelle (GO:0043226, 33,368 isoforms), and membrane part (GO:0044425, 33,251 isoforms) were the five most functional terms in the cellular component ontology. Within the molecular function category, binding (GO:0005488, 67,324 isoforms) was the largest group, followed by catalytic activity (GO:0003824, 63,247).

KEGG pathways database contains a systematic analysis of inner-cell metabolic pathways and functions of gene products. Metabolic pathway analysis of unique isoforms was also carried out using the KEGG annotation system. The results showed that 118,052 isoforms were classified into five main categories (**Figure S1D**). Most of isoforms included in the metabolism category were involved in many pathways, such as global and overview maps, and carbohydrate metabolism, amino acid metabolism, and energy metabolism. The second highly enriched category was genetic information processing, and the isoforms were mainly associated with translation and fold, sorting, and degradation. There were only 4976 isoforms included in environmental information processing and focused on the environmental adaptation pathways.

Moreover, all the isoforms were subjected to a search in the KOG database for functional prediction and classification. Overall, 119,842 isoforms were functionally classified into 25 specific categories (**Figure S1E**). Among them, general functional prediction only (35905 isoforms) was the largest category, followed by signal transduction mechanisms (17490 isoforms), post-translational modification, protein turnover, chaperone (12900 isoforms), function unknown (9480 isoforms), transcription (8785 isoforms), and carbohydrate transport and metabolism (8608 isoforms). Only 175 isoforms were assigned to cell motility.

### RNA-seq analysis

RNA samples from roots were individually prepared from non-waterlogging, waterlogging, and waterlogging recovery group plants and then sequenced on an Illumina instrument, to obtain comprehensive waterlogging and waterlogging recovery responses in *P. ostii*. Illumina sequencing data have been deposited in the Genome Sequence Archive under the accession number: CRA004511. In total, 377,050,830 raw reads were generated. After removing adaptor sequences, ambiguous nucleotides, and low-quality sequences, 337,972,250 clean reads were recorded. The lowest Q20 percentage and GC percentages were 96.89% and 44.51%, respectively, for the six libraries. The filtered clean reads were mapped to the reference transcriptome generated by PacBio ISO-seq. Of all the reads, 57.04–65.30% were mapped to the reference transcriptome, and 10.80–15.06% were uniquely mapped reads (**Table 2**).

**Table 2:** Information of RNA-seq raw data and clean reads mapped with the reference transcriptome.

Sample	Raw Reads	Clean Reads	Clean Bases (Gb)	Clean Reads Q20(%)	Clean Reads Q30(%)	GC (%)	Total Mapping (%)	Uniquely Mapping (%)
CK-1	51015062	46308650	6.95	97.41	92.92	47.58	62.37	15.06
CK-2	47056470	42587414	6.39	97.41	93.03	44.51	65.30	11.23
WT-1	85849198	76528162	11.48	96.89	92.00	44.73	63.18	11.20
WT-2	84681732	76237398	11.44	97.33	92.78	48.71	58.77	12.40
RT-1	54386540	48599822	7.29	98.05	94.47	45.55	63.16	12.31
RT-2	54061828	47710804	7.16	97.88	94.03	46.39	57.04	10.80
Sum	377050830	337972250	50.71					

### Identification and functional profiles of DEGs

**Figure 3:**
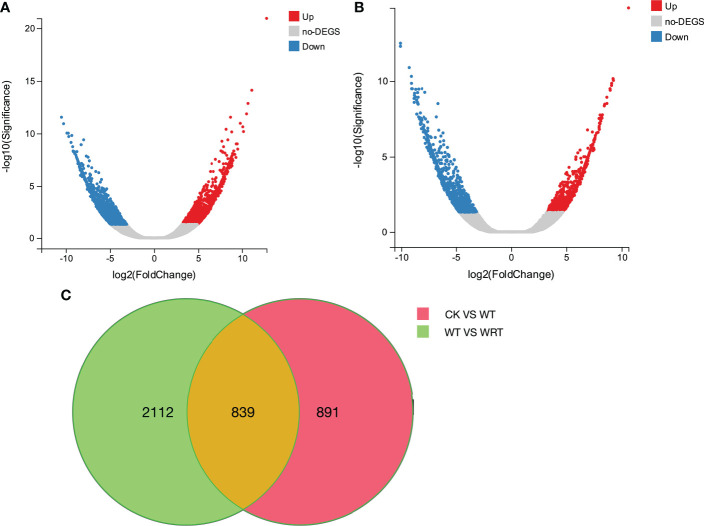
The distribution and expression levels of DEGs in *P. ostii* roots. **(A)**, Volcano plot of waterlogging treatment (CK VS WT); **(B)**, Volcano plot of waterlogging recovery treatment (WT VS WRT); **(C)**, Venn diagram of DEGs under waterlogging and its recovery treatment. The *x*-axis represents the log_2_ [Fold Change] values under the mean normalized expression of all isoforms (*y*-axis).

The expression levels of all isoforms were calculated using RSEM software and shown as FPKM values. DEGs were identified using DESeq2 with Q value (adjust P value) < 0.001 and fold change ≥ 2 or ≤ -2. A total of 2951 DEGs were obtained in response to waterlogging treatment (CK VS WT); 1358 DEGs were significantly upregulated, and 1593 DEGs were significantly downregulated (**Figure 3A**). As for the waterlogging recovery treatment (WT VS WRT), 1730 DEGs were observed, with 759 of them upregulated and 971 downregulated (**Figure 3B**).

**Figure 4:**
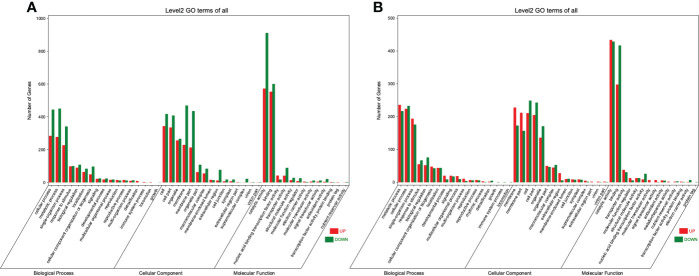
GO enrichment classification of DEGs in *P. ostii* roots. **(A)**, Waterlogging treatment (CK VS WT); **(B)**, Waterlogging recovery treatment (WT VS WRT).

The DEGs were then subjected to the Gene Ontology (GO) analysis to determine their functional classification. Under the WT, 2951 DEGs could be categorized into 45 functional groups on three main categories (biological process, cellular component, and molecular function) (**Figure 4A**). As for the WRT, these three main categories included 47 groups (**Figure 4B**). In both treatments, cellular process (GO:0009987), and metabolic process (GO:0008152) were the two largest groups enriched in the biological process; cell (GO:0005623), membrane (GO:0016020), membrane part (GO:0044425), and organelle (GO:0043226) were the four most prominent groups enriched in the cellular component; catalytic activity (GO:0003824) and binding (GO:0005488) were the two largest groups enriched in the molecular function. Using enrichment analysis, the top 20 significant enriched GO terms identified in the DEGs of two treatments (Q value ≤ 0.05) were shown in (**Figure S2**). Oxidoreductase activity (GO:0016491) was the most enriched term in both WT and WRT. In addition, there were five identical terms: heme-binding (GO:0020037), iron ion binding (GO:0005506), tetrapyrrole binding (GO:0046906), oxidoreductase activity, acting on paired donors, with incorporation or reduction of molecular oxygen (GO:0016705), and cofactor binding (GO:0048037) in Top 20 enriched terms of two treatments.

**Figure 5:**
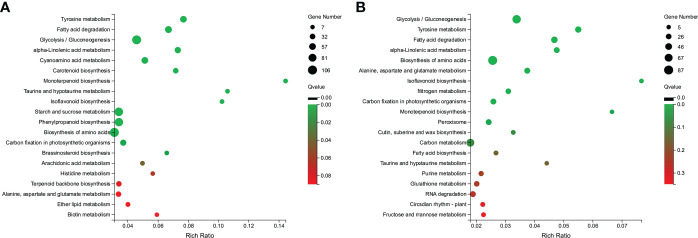
KEGG enrichment analysis of DEGs in *P. ostii* roots. **(A)**, Waterlogging treatment (CK VS WT); **(B)**, Waterlogging recovery treatment (WT VS WRT).

The DEGs were also subjected to KEGG analysis to investigate their biological functions further; 1404 DEGs of CK VS WT were assigned to 124 pathways, and 855 DEGs of WT VS WRT were categorized into 119 pathways. Enrichment analysis for DEGs showed that 15 pathways and 11 pathways were significantly enriched (Q value ≤ 0.05) under the WT (**Figure 5A**) and WRT (**Figure 5B**), respectively. Furthermore, 8 pathways, tyrosine metabolism (ko00350), fatty acid degradation (ko00071), alpha-linolenic acid metabolism (ko00592), glycolysis/gluconeogenesis (ko00010), monoterpenoid biosynthesis (ko00902), isoflavonoid biosynthesis (ko00943), biosynthesis of amino acids (ko01230), and carbon fixation in photosynthetic organisms (ko00710), were significantly enriched in both treatment groups. Interestingly, the proportion of upregulated and downregulated genes in these pathways behaved oppositely in the two treatment groups. For example, in the glycolysis/gluconeogenesis pathways, the number of upregulated genes was more than that of downregulated genes in the WT group. However, the number of downregulated genes was more than that of upregulated genes in the WRT group.

**Figure 6:**
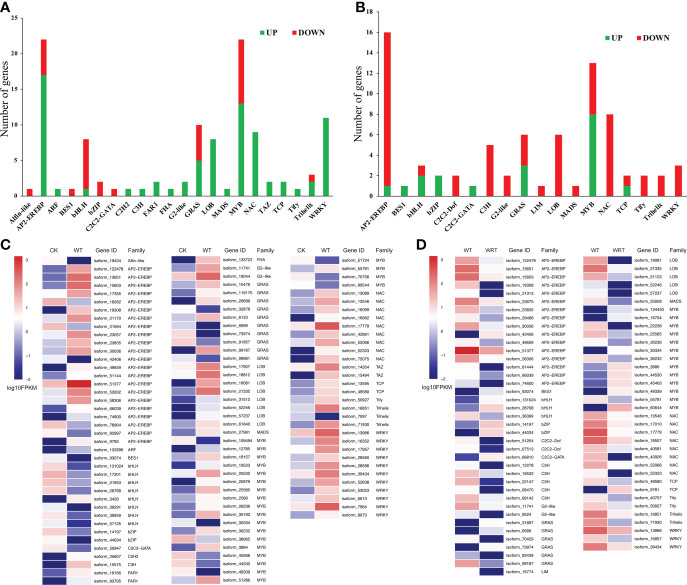
DEGs coding transcription factors based on their assigned protein families in *P. ostii* roots. **(A)**, Waterlogging treatment (CK VS WT); **(B)**, Waterlogging recovery treatment (WT VS WRT); **(C)**, Heatmap of DEGs coding transcription factors under waterlogging treatment; **(D)**, Heatmap of DEGs coding transcription factors under waterlogging recovery treatment.

Since transcription factors (TFs) could mediate the expression of genes involved in the waterlogging and waterlogging recovery, we analyzed the DEGs coding TFs. A total of 112 TFs were classified into 22 families based on their assigned protein in the WT group, which accounted for 3.80% of the DEGs. Of these, 80 TFs were upregulated, and 32 TFs were downregulated (**Figures 6A, C**). 76 TFs were classified into 18 families in the WRT group, which accounted for 4.40% of the DEGs. Among them, 19 TFs were upregulated and 57 TFs were downregulated (**Figures 6B, D**). Apetala 2/ethylene-responsive element binding protein (AP2/EREBP) and MYB family represented the most significantly expressed TFs (22) in the WT group, while the TFs belonging to AP2/EREBP (16) were the most significantly expressed in the WRT group, followed by the MYB family (13). On the whole, most of TFs were upregulated in the WT group but downregulated in the WRT group.

### Analysis of genes involved in key pathways

**Figure 7:**
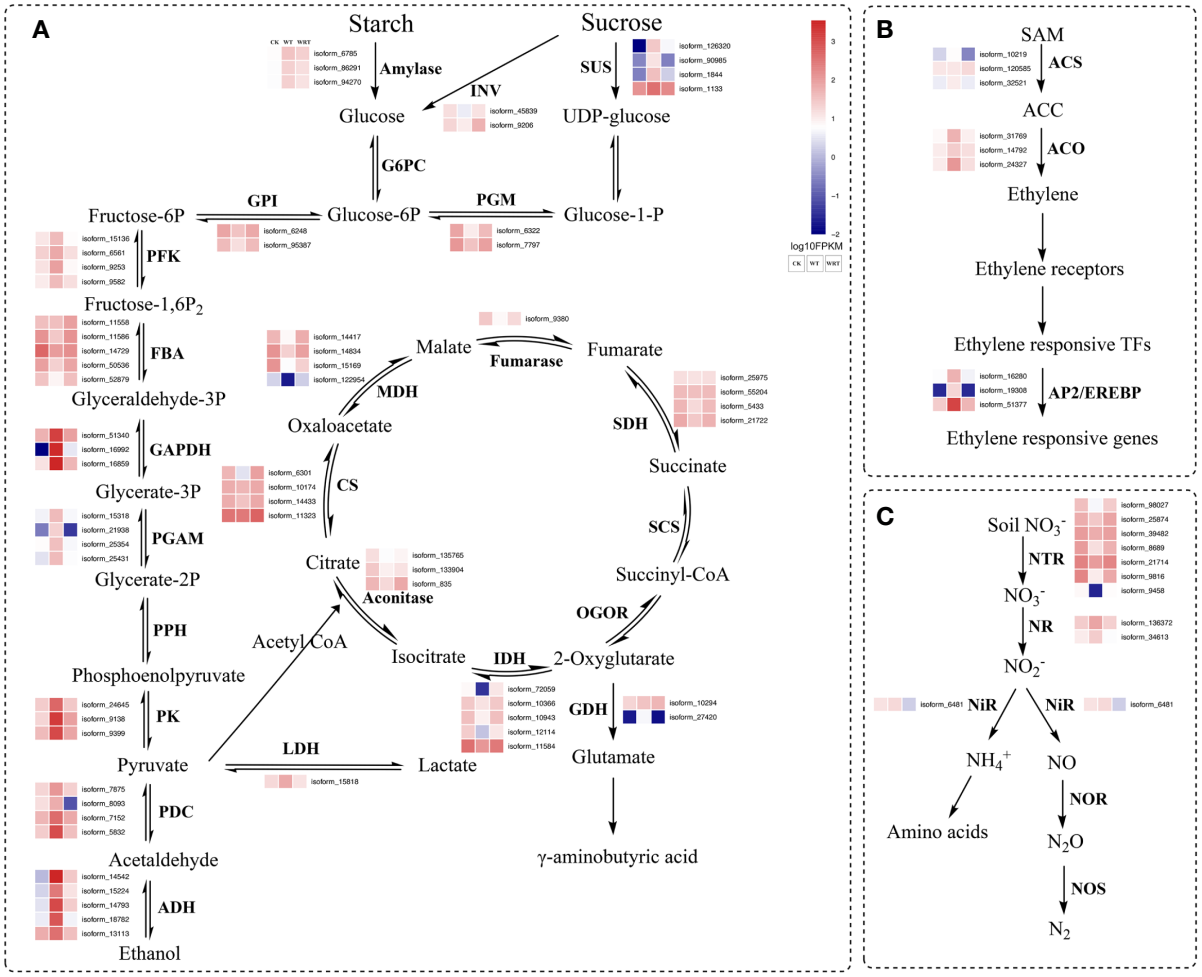
Waterlogging and waterlogging recovery caused genes encoding proteins involved in glycolysis, fermentation, citrate cycle **(A)**, ethylene biosynthesis **(B)**, nitrogen metabolism **(C)**. The red character represents DEGs in both CK VS WT and WT VS WRT. INV, invertase; SUS, sucrose synthase; G6PC, glucose-6-phosphatase; PGM, phosphoglucomutase; GPI, glucose-6-phosphate isomerase; PFK, 6-phosphofructokinase; FBA, fructose-bisphosphate aldolase; GAPDH, glyceraldehyde-3-phosphate dehydrogenase; PGAM, phosphoglycerate mutase; PPH, phosphopyruvate hydratase; PK, pyruvate kinase; PDC, pyruvate decarboxylase; ADH, alcohol dehydrogenase; LDH, lactate dehydrogenase; CS, citrate synthase; MDH, malate dehydrogenase; SDH, succinate dehydrogenase; SCS, succinyl-CoA synthetase; OGOR, 2-oxoglutarate/2-oxoacid ferredoxin oxidoreductase; IDH, isocitrate dehydrogenase; GDH, glutamate decarboxylase; ACS, 1-aminocyclopropane-1-carboxylate synthase; ACO, 1-aminocyclopropane-1-carboxylate oxidase; AP2/EREBP, apetala 2/ethylene-responsive element binding protein; NTR, Nitrate transporter; NR, Nitrate reductase; NiR, Nitrite reductase; NOR, Nitric-oxide reductase; NOS, Nitrous-oxide reductase.

**Figure 8:**
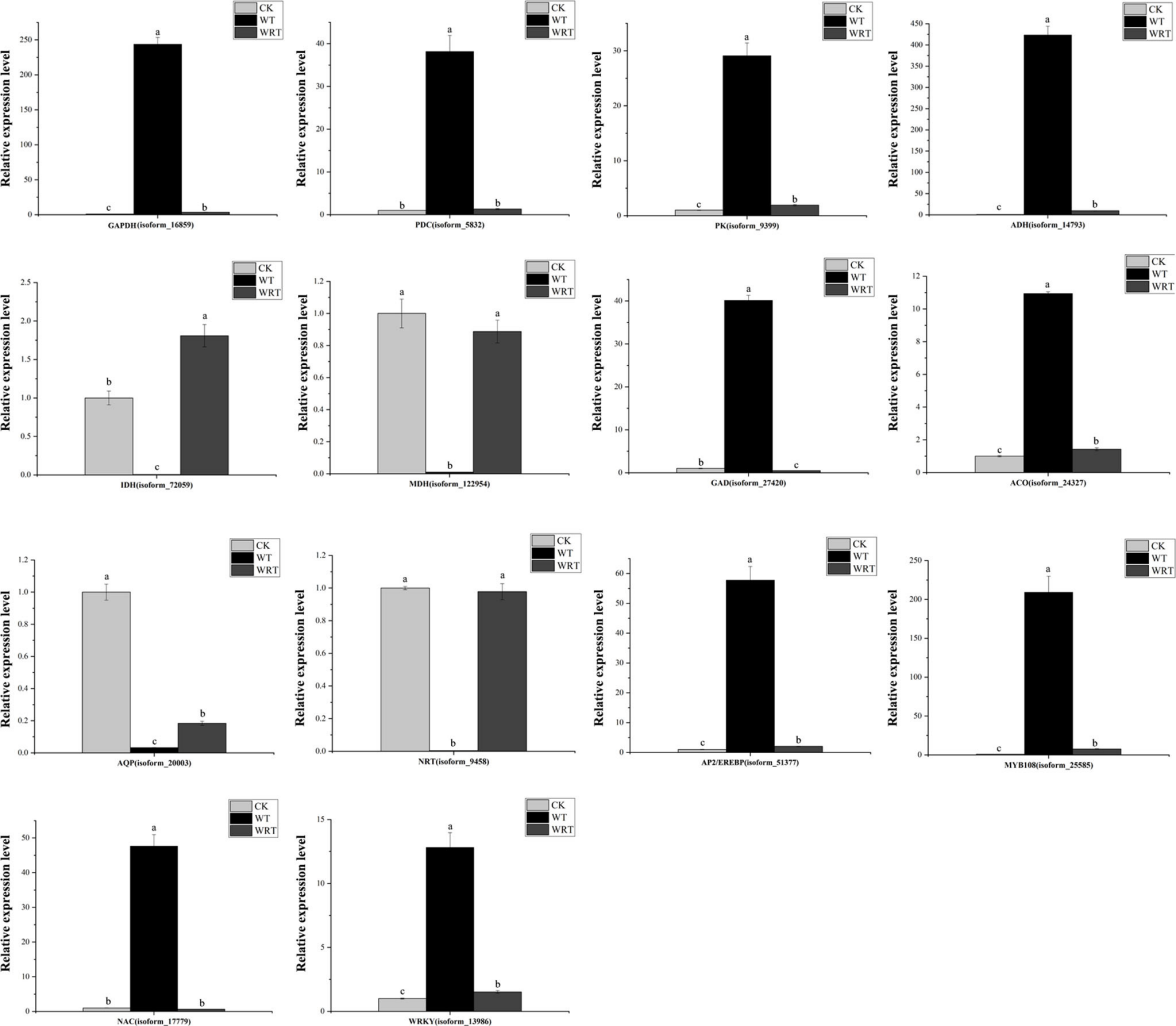
Quantitative real-time PCR validation of key genes. *Actin* was used as the reference gene. Expression values were normalized such that the expression levels of CK were set to 1. GAPDH, glycer-aldehyde-3-phosphate dehydrogenase; PK, pyruvate kinase; PDC, pyruvate decarboxylase; ADH, alcohol dehydrogenase; MDH, malate dehydrogenase; IDH, isocitrate dehydrogenase; GDH, glutamate decarboxylase; ACO, 1-aminocyclopropane-1-carboxylate oxidase; AQP, aquaporin; NRT, nitrate transporter. Values represent mean ± standard deviation (SD), and letters indicate significant differences according to Duncan’s multiple range test (*p* < 0.05).

The pathways of glycolysis, fermentation, citrate cycle, ethylene biosynthesis, and nitrogen metabolism were shown in **Figure 7**, and the RNA-Seq expression of transcripts encoding the enzymes involved in these pathways varied under WT and WRT. There were 839 DEGs in both the WT and the WRT (**Figure 3C**), and 14 DEGs that were mainly involved in glycolysis, fermentation, citrate cycle, ethylene biosynthesis, nitrogen metabolism, water absorption, and transcriptional regulation were selected for further qRT-PCR analysis (**Figure 8**). These DEGs encoded ADH, glyceraldehyde-3-phosphate dehydrogenase (GAPDH), PDC, pyruvate kinase (PK), isocitrate dehydrogenase (IDH), malate dehydrogenase (MDH), glutamate decarboxylase (GAD), aquaporin, nitrate transporter (NRT), 1-aminocyclopropane-1-carboxylate oxidase (ACO), WRKY, MYB, NAC, and AP2/EREBP, respectively. The expression levels of all fourteen isoforms significantly varied between the waterlogging and waterlogging recovery groups. Our results showed that IDH, MDH, aquaporin, and NRT were downregulated in the WT group, but upregulated in the WRT group. However, the changes of the other ten isoforms were opposite in the WT and WRT groups. Expression analysis of all the selected isoforms of a few genes was carried out using qRT-PCR to validate the sequencing results, which showed similar patterns to those observed in the RNA-Seq data.

## Discussion

Roots are the first organ to sense waterlogging, and hence play a key role in the waterlogging stress response (Langan et al., 2022). The root activity of *P. ostii* under waterlogging decreased significantly in comparison with the control, and increased as the waterlogging receded. Once a plant detects waterlogging, its priority is to reinstate the oxygen supply to the roots, which can be achieved by altering its root morphology and anatomy (Pedersen et al., 2021). These changes were also found in *P. ostii*. The tips of main roots were rotten, and a lot of fibrous roots fell off under waterlogging. Anatomic analysis showed that the cell size became larger due to a large amount of water absorption. This change in cell size may be due to the damage of cell membrane permeability. REC can reflect the degree of cell membrane damage (Zhao et al., 2019). After waterlogging, REC increased significantly, and then slightly decreased when the soil surface water was removed. These results indicated that waterlogging stress destroyed the cell membrane structure, and caused membrane permeability to become large, which was also supported by the anatomy data.

With advancements in technology, the studies on plant response to adverse abiotic stress are no longer limited to detecting the physiological characteristics but also identifying their molecular mechanisms. RNA-Seq has been successfully used to elucidate the response of plants to various environmental stresses, such as cold (Pang et al., 2013), salt (Postnikova et al., 2013), and drought (Zhao et al., 2019). Moreover, the combined hybrid approach of RNA-Seq and SMRT sequencing provides a more accurate integrated analysis of transcriptomes than using NGS alone (Chao et al., 2019). The clear advantage was that the traditional gene cloning to obtain full-length cDNA sequences, which is expensive, time-consuming, and inefficient, was not required (Deng et al., 2018). In the present study, we constructed the complete and accurate transcriptome of *P. ostii* roots using PacBio ISO-seq. Our study is novel because this is also the first full-length transcriptome sequencing of *P. ostii*.

In summary, we constructed three libraries (inserted size 1–2 kb, 2–3 kb, and > 3 kb) and obtained 4.71, 4.6, and 4.49 GB of raw data, respectively. A total of 13.8 GB of raw data was generated, and 187,564 full-length isoforms with an average length of 2323 bp were mapped to seven databases (NR, NT, KEGG, GO, KOG, SwissProt and Pfam). These data were similar to the full-length transcriptome profiling for flower bud of *P. suffruticosa* ‘High Noon’ (Chang et al., 2019) and we highlight how the gene sequence information would be instrumental for the further study of tree peony. The mRNAs of six *P. ostii* roots, belonging to CK, WT, and WRT groups, were sequenced using RNA-Seq and referred to as the transcriptome generated by ISO-Seq. We identified 2951 DEGs and 1730 DEGs in response to WT and WRT, respectively, and 839 DEGs were common between the two treatment groups. Our study demonstrated that the mechanisms involved in the WRT group were the reverse of the WT group and had some unique response mechanisms. Moreover, we identified response mechanisms involved in the WT group that did not manifest in the WRT group. Previous studies solely focused on waterlogging (Lee et al., 2014; Ren et al., 2017) or waterlogging recovery (Yeung et al., 2018). However, in our study, the gene expression differences underlying these two adverse stress conditions were investigated simultaneously, with the aim to deepen the comprehensive understanding of this sequential stress.

In the condition of waterlogging stress, plants have two response mechanisms. Hypoxia escape syndrome enables plants to overcome waterlogging through the elongation of petioles, enables stems, and leaves to get more oxygen through the formation of large lenticels, adventitious roots, and aerenchyma facilitates oxygen transportation and storage. However, these processes require much higher ATP and may lead to their death (McDonald et al., 2002; Glenz et al., 2006). The other mechanism is hypoxic resting syndrome, where the glycolysis and fermentation processes are accelerated to maintain the energy level, while the tricarboxylic acid cycle (TCA) is inhibited (Bailey-Serres and Voesenek, 2008; Mustroph et al., 2010; Bailey-Serres et al., 2012; Pan et al., 2021). Several life processes, such as cell differentiation and growth, protein synthesis, and cell wall formation, are also slowed down. Plants can survive this energy crisis by reducing energy consumption (Voesenek and Bailey-Serres, 2015). In our previous study, the morphological characteristics of *P. ostii* did not change under waterlogging stress (Hu et al., 2017). In the present study, the genes encoding ADH, GAPDH, PDC, and PK involved in glycolysis and fermentation were significantly upregulated under waterlogging as identified by RNA-Seq and qRT-PCR verification.

We also found that the genes encoding IDH and MDH involved in TCA cycle were significantly downregulated under waterlogging stress. Moreover, most DEGs involved in carotenoid biosynthesis, monoterpenoid biosynthesis, starch and sucrose metabolism, phenylpropanoid biosynthesis, and isoflavonoid biosynthesis were downregulated. Our results supported the longstanding notion that plants respond to waterlogging stress by regulating energy production and consumption (Bailey-Serres et al., 2012). As for the waterlogging recovery treatment, the altered gene expression trends mentioned above were opposite to waterlogging treatment. All these results collectively indicated that the strategy of *P. ostii* adopted hypoxic resting syndrome in response to WT.

Hypoxia caused by waterlogging stress modulates the carbon metabolism involved in energy production, consumption, and nitrogen metabolism (Ren et al., 2017). Nitrate reductase and nitrite reductase are two critical enzymes involved in the regulation metabolism, and they can convert nitrate in plants to nitric oxide (Juntawong et al., 2014). However, in the present study, the genes of these two enzymes were not differentially regulated in WT and WRT groups. We also found that the gene encoding nitrate transporter was significantly downregulated under WT and upregulated in response to WRT, as verified by qRT-PCR. Nitrate transporter is responsible for nitrate uptake by plant roots from the soil, its transport, and intracellular redistribution in plants (Jia et al., 2014). We hypothesized that the roots of *P. ostii*, under waterlogging stress, reduce the production of endogenous nitric oxide by decreasing nitrate uptake from the soil, which is important for avoiding the damage caused by a higher concentration of nitric oxide in the plants.

Aquaporins, proteins belonging to Membrane Intrinsic Proteins (MIP) family facilitate the bidirectional transport of water through biological membranes (Martinez-Ballesta et al., 2014). Enabling enhanced water absorption of roots is one of the main mechanisms for maintaining water content under adverse conditions. Aquaporins play a major role in regulating the hydraulic conductivity that ultimately affects the water uptake capacity of plants (Chaumont and Tyerman, 2014). Numerous studies have been conducted to elucidate the important roles of aquaporins in several plant species in response to salt and drought stresses (Kadam et al., 2017), while there is a severe lack of adequate attention to aquaporins in response to waterlogging stresses, especially for *Paeonia* species. The gene encoding TIP1-3 was significantly downregulated in the WT group, followed by a significant increase after WRT, although it did not return to the level of the CK group. In sorghum roots, the observed initial upregulation of aquaporin gene expression in response to short-term exposure (18 h) to stress may enhance water uptake to maintain the plant water status, while reduced expression after prolonged exposure (96 h) to stress may reduce hydraulic conductivity (Lv et al., 2016). In this study, *P. ostii* suffered a long-term waterlogging (3 d), and the water content in the roots was nearly saturated. Hence, the aquaporins gene expression naturally decreased to reduce the water intake, which was in line with the above result. After 7 d of waterlogging recovery, the aquaporin gene was significantly upregulated, but it did not reach the level of CK, which could be attributed to *P. ostii* still being in the recovery phase.

Ethylene, an important plant hormone, mediates tolerance and adaption of plants to adverse abiotic stresses including waterlogging (Lv et al., 2016). In the ethylene biosynthesis pathway, S-adenosylmethionine is used as a substrate to generate 1-aminocyclopropane-1-carboxylate (ACC) under the catalysis of ACC synthase (ACS), and then ACC gets oxidized by ACC oxidase (ACO) to ethylene. Therefore, ACS and ACO are two key enzymes controlling ethylene biosynthesis in plants (Zhang et al., 2018b). Under the WT condition, the expression of *ACO* in the root of *P. ostii* was significantly upregulated as verified by qRT-PCR, and downregulated in response to the WRT condition. Unexpectedly, *ACS* was not a DEG in response to WT and WRT conditions, which may be because the production of ACC did not take place in the roots of *P. ostii*. In contrast to our results, *ACS* expression was significantly upregulated, while *ACO* was not expressed differentially in the waterlogged *Rehmannia glutinosa* roots (Wang et al., 2017). This observation indicated that the oxidation of ACC to ethylene was not in the roots of *R. glutinosa*, and hence the difference of ethylene production in response to waterlogging stress between different plant species.

Transcription factors (TFs) are master regulators of abiotic stress responses in plants. These TFs probably initiate the indirect-late phase of responses by binding to *cis*-acting elements in the promoters of specific target genes encoding proteins with specific functions (Lindemose et al., 2013). In the present study, 3.80% and 4.40% of the DEGs encoding TFs were identified in the two treatments respectively, indicating that transcriptional regulation played an important role in response to WT and WRT conditions. Consistent with the previous finding that ethylene synthesis and perception were activated (Juntawong et al., 2014), our study recognized AP2/EREBP as the most pronounced TFs in response to both WT and WRT. Their expression patterns were closely followed by the MYB TFs, which were regarded as the second largest TF family in response to WT and WRT. Some studies have reported that MYB TFs played a critical regulatory role in response to waterlogging stress of many plants (Wang et al., 2017; Li et al., 2018) and various stress of *P. ostii* (Gai et al., 2013; Wang et al., 2016). bHLH and WRKY TFs were expressed more in response to WT, but less in WRT, which suggested that these two TFs may play a more critical role in waterlogging stress. Four TFs genes encoding AP2/EREBP, WRKY, MYB, and NAC were chosen for further expression analysis by qRT-PCR due to their high differential expression as captured in the sequencing results. They were all induced in response to the WT condition, while the decreasing expression levels were observed under the WRT condition. These results indicated that all the validated TFs were highly responsive to waterlogging and waterlogging recovery. These TFs need to be analyzed further to identify their target genes, which may thus provide important insights into the molecular mechanisms underlying waterlogging tolerance in *P. ostii*.

In summary, this study was the first report on a comprehensive physiological and transcriptomic analysis of *P. ostii* roots in response to waterlogging and its recovery. The strategy of *P. ostii* adopted hypoxic resting syndrome in response to waterlogging. A hybrid approach combining SMRT and NGS sequencing platforms was applied in the present study, and the full-length transcriptome sequencing of *P. ostii* was reported for the first time. A lot of responsive transcripts and genes that might play an important role in waterlogging and its recovery of *P. ostii* were identified, especially those involved in the glycolysis and fermentation, tricarboxylic acid cycle, nitrogen metabolism, water absorption, ethylene biosynthesis, and transcriptional regulation. The detailed characterization of individual genes must be performed as the next step to clarify their specific functions. These results will enhance our understanding of defense mechanisms against waterlogging stress in *P. ostii*, and the full-length transcriptome established in the present study will provide the data support for future studies on *P. ostii* at the molecular levels.

## Data availability statement

The datasets presented in this study can be found in online repositories. The name of the repository and accession number are as follows: Genome Sequence Archive, CRA004511.

## Author contributions

JY and YH conceived the project. JY designed all the experiments. JY, XZ, and XL analyzed the data. XZ and MZ collected the samples and performed the experiments. XZ, JY, and YH drafted and revised the manuscript. All authors read and approved the final version of the manuscript.

## Funding

This research was funded by Shanghai Science and Technology Committee (14JC1403902, 21DZ1202000), Special Fund for Scientific Research of Shanghai Landscaping Administration Bureau Program (G222415), National Natural Science Foundation of China (31470328, 32000240), Open Project of Shanghai Key Laboratory of Plant Functional Genomics and Resources (PFGR202203), and Key project at the central government level: The ability establishment of sustainable use for valuable Chinese medicine resources (2060302).

## Acknowledgments

We thank the joint Postgraduate Shaobo Du, Yanli Hu, Yunpeng Wang, and Yanan Xuan for collecting samples.

## Conflict of interest

The authors declare that the research was conducted in the absence of any commercial or financial relationships that could be construed as a potential conflict of interest.

## Publisher’s note

All claims expressed in this article are solely those of the authors and do not necessarily represent those of their affiliated organizations, or those of the publisher, the editors and the reviewers. Any product that may be evaluated in this article, or claim that may be made by its manufacturer, is not guaranteed or endorsed by the publisher.
