# Supplementary material for: PacBio full-length sequencing integrated with RNA-seq reveals the molecular mechanism of waterlogging and its recovery in Paeonia ostii
Source: Front Plant Sci. 2022 Nov 3;13:1030584. doi: 10.3389/fpls.2022.1030584 (PMC9669713; doi:10.3389/fpls.2022.1030584)
Supplement: Supplementary file 1 [file DataSheet_1.docx]

Supplementary Material

**
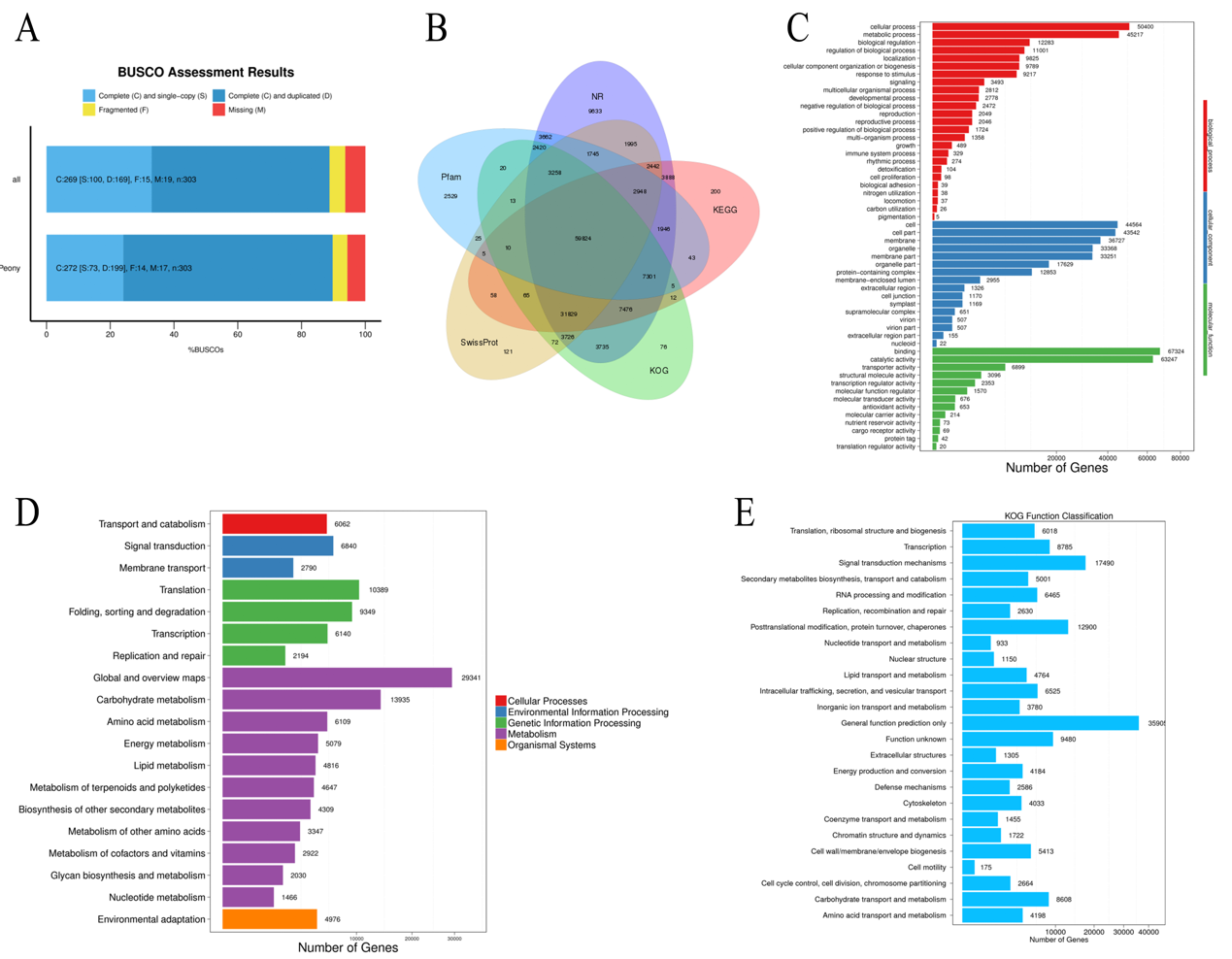
**

**Supplementary Figure 1.** Summary of Single-molecule real-time sequencing analysis. A, BUSCO assessment results; B, Venn diagram of all isoforms from the reference transcriptome hits of KOG, NR, Kyoto Encyclopedia of Genes and Genomes (KEGG), SwissProt, and Pfam databases; C, Gene ontology classification of all identified isoforms from the reference transcriptome; D, KEGG classification of all identified isoforms from the reference transcriptome; E, KOG classification of all identified isoforms from the reference transcriptome.

**
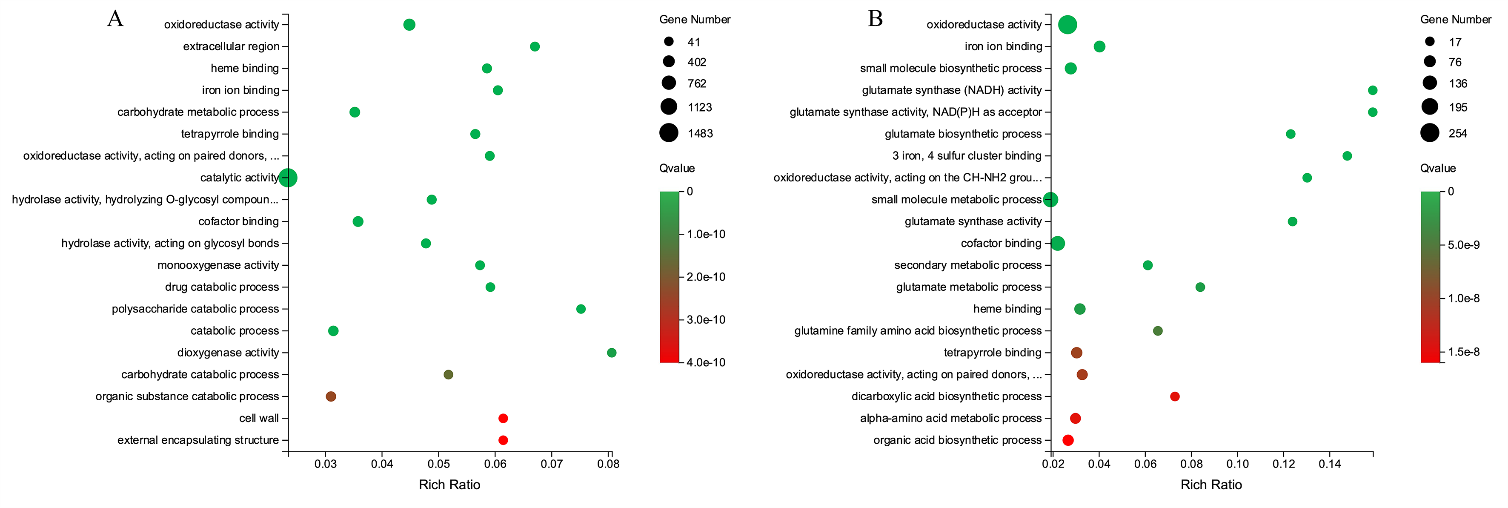
**

**Supplementary Figure 2.** Gene Ontology (GO) enrichment analusis of differentially expressed genes (DEGs) in *P. ostii* roots. A, Waterlogging treatment (CK VS WT); B, Waterlogging recovery treatment (WT VS WRT).

**Supplementary** **Table 1.** Gene-speciﬁc primer sequences for detection by qRT-PCR.

| Gene ID | Gene annotation | Forward primer (5'-3') | Reverse primer (5'-3') |
| --- | --- | --- | --- |
| isoform_16859 | GAPDH | GCTGCTTCCTTCAACATCATT | TGAGATCCACGACTGAAACAT |
| isoform_5832 | PDC | CTGCAATTCCTCATCCAACAT | GTTGTGCTTTAGCTACCCTTA |
| isoform_9399 | PK | CCTTGATTATGGTGACGTGTT | TGTATTTTGCCACCAGCTTTG |
| isoform_14793 | ADH | CCCTAAGAGATTTGATGAAGC | GAACACACTCAAATGCAGACA |
| isoform_72059 | IDH | AGGATTTGGTTCTTTGGGACT | CCTCGTGTCCATGCAAATATT |
| isoform_122954 | MDH | TGCTAACATGTGCTTCAAGGT | TCGTCTCACAAATGTCAGGAA |
| isoform_27420 | GDH | GATCAACCCACTTTTACCCTT | CAGTCTTCACCAATCCTTCTT |
| isoform_24327 | ACO | TTATCTGAAGAAAACCCACCA | TTTGAAATACTCGAATCCAGA |
| isoform_20003 | AQP | TTCAGTCGGTGCAAACATTTC | GGATAGAGCAAATGCAGAAGT |
| isoform_9458 | NRT | TGGATCTCTTTCTTCACTTGC | ATGACCAGCCTAGAGAAAATG |
| isoform_51377 | AP2/EREBP | CGCAAGATCAGAGGAAAGAAA | GATATATCAACCCCGTAAGAC |
| isoform_25585 | MYB | GGTCCAAGATTGCACAACATT | GCATCCAACAAGTCCTAATCA |
| isoform_17779 | NAC | GAATATCGCCTCGCAAATGTT | TCCAGGCTTTTGATCGAATGT |
| isoform_13986 | WRKY | ATAACCGGTGAGACTGATAGA | ACCCTTTTCACCATCGATTTC |
| isoform_19040 | *β*-actin | GCTCTCCTTAGCCTCTTGCC | AAGGTGACCCTCAGCAGTTG |

**Supplementary Table** **2.** Information of polymerase reads generated by the PacBio Sequel platform.

| Library | Total Reads | Total Base (GB) | MaxLength (bp) | MeanLength (bp) | N50 Length(bp) |
| --- | --- | --- | --- | --- | --- |
| 1-2k_161222 | 694 | 0.01 | 44417 | 11249.33 | 20880 |
| 1-2k_161228 | 138357 | 1.99 | 51618 | 14386.63 | 26122 |
| 1-2k_170104 | 12207 | 0.18 | 48971 | 14402.71 | 26159 |
| 1-2k_170111 | 19029 | 0.29 | 51253 | 15122.7 | 27504 |
| 1-2k_170305 | 206227 | 2.24 | 75930 | 10868.51 | 19338 |
| 2-3k_161222 | 41063 | 0.48 | 47855 | 11691.64 | 23255 |
| 2-3k_170104 | 42266 | 0.57 | 46330 | 13465.51 | 24755 |
| 2-3k_170111 | 40607 | 0.52 | 47186 | 12895.8 | 24293 |
| 2-3k_170313 | 5583 | 0.07 | 49559 | 12469.38 | 24768 |
| 2-3k_170322 | 316906 | 2.96 | 63100 | 9355.7 | 18927 |
| 3k_131223 | 450017 | 4.49 | 60687 | 9984.57 | 18158 |

**Supplementary** **Table 3.** Information of Reads of Insert.

| Library | Reads of Insert | Mean Read Length of Insert (bp) | Mean Read Quality of Insert | Mean Number of Passes |
| --- | --- | --- | --- | --- |
| 1-2k_161222 | 600 | 1835 | 0.91 | 6 |
| 1-2k_161228 | 123534 | 1544 | 0.94 | 9 |
| 1-2k_170104 | 11325 | 1582 | 0.94 | 8 |
| 1-2k_170111 | 17798 | 1419 | 0.94 | 10 |
| 1-2k_170305 | 189106 | 1403 | 0.93 | 6 |
| 2-3k_161222 | 36430 | 2570 | 0.9 | 3 |
| 2-3k_170104 | 37880 | 2637 | 0.92 | 4 |
| 2-3k_170111 | 36545 | 2201 | 0.92 | 4 |
| 2-3k_170313 | 5203 | 2004 | 0.92 | 5 |
| 2-3k_170322 | 265161 | 2144 | 0.89 | 3 |
| 3k_131223 | 331876 | 3265 | 0.87 | 2 |

**Supplementary** **Table 4.** Information of full-length non-chimeric reads.

| Library | Classifyed ROI | Full-length non-chimeric reads | Mean Read Quality | Mean Number of Passes | Mean full-length non-chimeric read length (bp) |
| --- | --- | --- | --- | --- | --- |
| 1-2k_161222 | 348 | 362 | 0.96 | 9 | 1172 |
| 1-2k_161228 | 87467 | 98087 | 0.97 | 10 | 1223 |
| 1-2k_170104 | 8184 | 9726 | 0.97 | 10 | 1211 |
| 1-2k_170111 | 12808 | 13962 | 0.97 | 12 | 1196 |
| 1-2k_170305 | 119422 | 132349 | 0.97 | 9 | 1215 |
| 2-3k_161222 | 17639 | 18853 | 0.97 | 5 | 2397 |
| 2-3k_170104 | 21844 | 23528 | 0.97 | 6 | 2389 |
| 2-3k_170111 | 20132 | 20706 | 0.97 | 7 | 2145 |
| 2-3k_170313 | 2394 | 2428 | 0.98 | 7 | 2041 |
| 2-3k_170322 | 112551 | 115036 | 0.97 | 5 | 2188 |
| 3k_131223 | 111355 | 111891 | 0.95 | 4 | 3562 |

**Supplementary** **Table 5.** Information of unique full-length isoforms.

| Total_number | average_length | N50 | N90 | Max_length | Min_length | Sequence_GC (%) |
| --- | --- | --- | --- | --- | --- | --- |
| 187564 | 2323 | 2722 | 1311 | 14860 | 200 | 40.69% |
